# Harnessing the power of an advanced *in vitro* 3D liver model and error-corrected duplex sequencing for the detection of mutational signatures

**DOI:** 10.1093/mutage/geaf015

**Published:** 2025-08-06

**Authors:** Gillian E Conway, Bérénice Chavanel, François Virard, Ume-Kulsoom Shah, Michael J Burgum, Stephen J Evans, Michael Korenjak, Laura E Thomas, Gareth J Jenkins, Jiri Zavadil, Shareen H Doak

**Affiliations:** In Vitro Toxicology Group, Institute of Life Science, Swansea University Medical School, Swansea, Wales SA2 8PP, United Kingdom; Epigenomics and Mechanisms Branch, International Agency for Research on Cancer, World Health Organization, Lyon 69007, France; Epigenomics and Mechanisms Branch, International Agency for Research on Cancer, World Health Organization, Lyon 69007, France; Cancer Research Center, INSERM U1052–CNRS UMR5286, Centre Léon Bérard, University Claude Bernard Lyon 1, Lyon 69008, France; In Vitro Toxicology Group, Institute of Life Science, Swansea University Medical School, Swansea, Wales SA2 8PP, United Kingdom; In Vitro Toxicology Group, Institute of Life Science, Swansea University Medical School, Swansea, Wales SA2 8PP, United Kingdom; In Vitro Toxicology Group, Institute of Life Science, Swansea University Medical School, Swansea, Wales SA2 8PP, United Kingdom; Epigenomics and Mechanisms Branch, International Agency for Research on Cancer, World Health Organization, Lyon 69007, France; In Vitro Toxicology Group, Institute of Life Science, Swansea University Medical School, Swansea, Wales SA2 8PP, United Kingdom; In Vitro Toxicology Group, Institute of Life Science, Swansea University Medical School, Swansea, Wales SA2 8PP, United Kingdom; Epigenomics and Mechanisms Branch, International Agency for Research on Cancer, World Health Organization, Lyon 69007, France; In Vitro Toxicology Group, Institute of Life Science, Swansea University Medical School, Swansea, Wales SA2 8PP, United Kingdom

**Keywords:** next-generation sequencing, duplex sequencing, in vitro, liver, 3D models, genotoxicity

## Abstract

Genotoxicity testing plays a crucial role in evaluating the hazards posed by various chemicals. Traditional methods, such as the Ames test, mammalian cell mutation assays and the transgenic rodent assay have certain limitations, including laborious procedures and/or reliance on animal models. The aim of this study was to determine the potential of using error-corrected next-generation sequencing, specifically duplex sequencing (DS), as an alternative method for the detection of point mutations in conjunction with advanced *in vitro* models. This study establishes an easy to use, adaptable *in vitro* 3D HepG2 model, that shows good viability and liver functionality over 14 days. 3D HepG2 spheroids were exposed to aristolochic acid in a repeated dose regime over 4 days. This was shown to significantly induce micronucleus formation, indicative of fixed DNA damage, in a dose-dependent fashion. DS coupled with mutational signature analyses revealed a predominant treatment-specific T:A > A:T-enriched mutational signature explained by COSMIC signature SBS22 derived from human cancers associated with aristolochic acid exposure. *De novo* extraction provided a stable signature, of which > 40% were unambiguously explained by SBS22. These results demonstrate that the presented 3D HepG2 spheroid model is appropriate for assessing chemically induced fixed DNA damage. Additionally, we provide evidence that DS applied to the studied *in vitro* 3D model has the capacity to reveal specific mutational signatures of mutagenic exposures. The modern integrative approach will improve the understanding of mechanisms of carcinogenesis related to chemical exposures by providing a cost-effective and efficient means to assess genotoxicity and mutagenicity. With the inclusion of mutational signature analyses, this approach would see a reduction in reliance on animal models and the enhancement of hazard assessment accuracy.

## Introduction

Testing for genotoxicity, one of the key characteristics of carcinogens (KCCs) [1], is a fundamental component of the hazard assessment of chemical agents. This involves *in vitro* tests that are capable of quantifying fixed DNA damage, including point mutations, structural chromosomal damage, and numerical changes in chromosome number*.* The current standard for *in vitro* approaches to point mutation detection involves the bacterial reverse mutation (Ames) test [Organisation for Economic Co-operation and Development (OECD) TG 471] and/or *in vitro* mammalian cell gene mutation tests (e.g. using the *HPRT* and *XPRT* OECD TG 476). These methods are sensitive, but not without limitations. OECD TG 476 provides guidance on a number of applicable cell lines; however, all of them are rodent cell lines and are p53 deficient. The *HPRT* assay is lengthy and laborious, and the Ames test uses mutant strains of bacteria, limiting comparison with mammalian cells. Additionally, the *HPRT* assay can only be performed in select cell lines using a 2D systems approach. Some of the constraints associated with those assays can be overcome by using advanced *in vitro* culture systems. However, a limitation of these advanced systems for hazard assessment is the inability to assess point mutations, instead measuring only chromosomal damage, typically through the *in vitro* micronucleus test (OECD TG 487). These limitations may be overcome by development and refinement of current test systems. One such emerging approach involves error-corrected next-generation sequencing (ecNGS), i.e. duplex sequencing (DS) techniques. DS can reliably measure DNA damage and could also reduce the need for animal models and for reporter genes for point mutation testing.

There has been significant focus on the development and implementation of advanced omics-based technologies that can increase the efficiency and specificity of currently used hazards assessment tests. This need has been most recently summarized in a study assessing the end points for evaluating mechanistic evidence of carcinogenic hazards using the KCC framework [2]. Next-generation sequencing (NGS) has transformed mutation detection by facilitating extensive high-output, genome-wide analysis, enabling characterization of mutational signatures specific to chemical exposures and wherever applicable, the functional impact on cancer relevant genes [3]. However, there remains a high technical error rate for standard NGS (~ 1 × 10^−3^) making distinguishing true somatic mutations from sequencing artefacts difficult [4], unless clonal cell populations are sequenced. Hence, genome sequencing-based mutation analysis using *in vitro* models, has long been limited to a stable, clonal cell population, the generation of which is an extensive and lengthy process [5]. The development of technologies like DS [6], a novel DNA-based ecNGS approach, has the capacity to revolutionize mutagenicity testing. One significant advantage is the reduction in the background error rate from 1 × 10^−3^ to 1 × 10^−7^ [4, 7]. Coupled with advanced data analysis approaches, DS can directly detect, quantify, and characterize mutations in heterogeneous cell populations [8, 9]. Recent studies have demonstrated that DS can successfully measure increased mutation frequency (MF) in both mouse and rat *in vivo* following exposure to different carcinogens [7, 10–13].

In 2004, the Catalogue of Somatic Mutations in Cancer (COSMIC) database was established to store somatic mutation data in a dedicated location [14], and since 2013 it contains somatic mutational signatures extracted from thousands of cancer genomes and exomes, including suggested aetiologies related to human cancer [15, 16]. In the last decade, there has been substantial focus on using NGS approaches to model mutational signatures in both *in vitro* and *in vivo* models following exposure to environmental carcinogens, in an effort to better understand the aetiology of human cancer [17–25]. It has become evident that certain genotoxic carcinogens cause very specific mutational signatures, which can be used to determine their mechanism of action. Mutational signatures characterized using experimental models can then be compared to the mechanism-associated mutational signatures extracted from sequenced human cancer and normal genomes [12, 26, 27] or established as *de novo* signatures experimentally associated with a specific exposure. The effort can be scaled up as attested by a compendium of mutational signatures derived from exposure of human-induced pluripotent stem cells to 79 different suspected or known carcinogens [26], and a comprehensive catalogue of experimentally derived signatures is now available in the COSMIC [28] and the Signal [29] databases. ecNGS approaches such as DS have demonstrated the sensitivity and accuracy required to directly measure genotoxin-induced DNA damage. This will help circumvent certain challenges associated with the traditionally used genotoxicity assessment methods.

Two-dimensional (2D) *in vitro* models have been the benchmark for decades; however, it is now widely accepted that 2D systems have several limitations and do not adequately simulate an *in vivo* environment [30, 31]. Therefore, in keeping with the principles of the 3Rs, to reduce, replace, and refine animal testing, there is now extensive research on the development and validation of advanced *in vitro* models that better mimic human organ systems, to offer viable alternatives to *in vivo* and 2D *in vitro* test systems. This is also supported by international bodies such as the OECD [32]. Hepatic toxicology is fundamental when considering chemical/drug hazard assessment, as the liver is essential for maintaining metabolic homeostasis and detoxification of both endogenous and exogenous substances. Furthermore, 3D liver cultures have been shown to better mimic the structural and functional complexity of the liver *in vitro* [33]*.* An advanced 3D HepG2 *in vitro* liver model has been developed that demonstrates good hepatic function and metabolic activity compared to 2D systems supporting multiple exposure regimes (both acute and long-term, single-dose exposures and repeated dose exposures) which can be applied to either single or co-culture 3D liver spheroid models [34–36]. This model has also been successfully adapted for use with the *in vitro* micronucleus assay [37] demonstrating its efficacy as a model that can be used for genotoxicological assessment.

The integration of ecNGS technologies, i.e. DS, with 3D cell culture systems could address many of the limitations of current testing systems and thus revolutionize toxicological research, particularly in the study of chemical hazard assessment. The aim of this study was to investigate whether our advanced *in vitro* 3D liver model can be used for detection of fixed DNA damage, using the gold standard micronucleus assay and ecNGS/DS to detect point mutations and a single-base substitution (SBS) mutational signature corresponding to the effects associated with exposure to the known mutagenic agent aristolochic acid (AA).

## Materials and methods

### Cell culture

The Human Caucasian Hepatocellular Carcinoma-derived epithelial cell line, HepG2 (ATCC HB-8065) monolayers were cultured in Dulbecco’s Modified Eagle Medium with 4.5 g/L D-glucose and L-glutamine (GIBCO, Paisley, UK) supplemented with 10% fetal bovine serum and 1% penicillin/streptomycin antibiotic (GIBCO, Paisley, UK). HepG2 cells were sub-cultured every 3–5 days, once 80% confluency was reached. To prepare spheroids, cells were trypsinized (0.05% trypsin/EDTA solution; GIBCO, Paisley, UK) and resuspended in media. 3D spheroids were formed by adding 100 ul of cell suspension (concentration: 40 000 cells/ml) to each well of a 96-well ultra-low attachment plate (Nunclon™ Sphera™, ThermoFisher Scientific) at 4000 cells per spheroid/well. To ensure enough cells were recovered for each endpoint, one plate (96 pooled spheroids) per concentration and endpoint was utilized. Cells were incubated at 37°C and 5% CO_2_ atmosphere for 48 h to form spheroids prior to treatment. Cells were then treated with either dimethyl sulfoxide (vehicle control) or AA at a final concentration of 5–20 μM for 48 h after which the cells were treated for a second time with AA or vehicle control and left for an additional 24 h prior to harvesting for downstream analysis. Aflatoxin B1 was used as a positive control for the micronucleus assay.

### Cell viability

Cell viability of the spheroids was assessed using the cell exclusion assay, using erythrosin b stain (Labtech, UK). Spheroids were pooled (96 spheroids) and centrifuged at 230 g for 5 min. To remove any residual media, the cell pellet was re-suspended in 1 ml phosphate buffered saline (PBS) (GIBCO, Paisley, UK) and centrifuged at 230 g for 5 min. To dissociate the spheroids, the cell pellet was re-suspended in trypsin/EDTA for 8–10 min. Fresh media was added in equal volumes to neutralize the trypsin. The cells were centrifuged at 230 g for 5 min and re-suspended in fresh media. Cell viability was then analysed using 1:1 ratio of erythrosin b on the Luna II automated cell counter [38] and the relative viable cell count calculated.

### Micronucleus assay

The remaining pooled cells were washed once in PBS, fixed, and slides prepared as previously described [34, 37, 39]. The cell density on the slide was examined using the Olympus BH2 microscope at ×100 objective and left to dry. Prior to automated scoring using the Metafer system, 30 μl of Vectashield Mounting Medium with 4′,6-diamidino-2-phenylindole (Vector Laboratories, Peterborough, UK) was added and a coverslip applied. When scoring, detection of micronuclei (Mn) in mononucleated cells was performed as previously described by Manshian *et al.* and Chapman *et al.* [39, 40]. A minimum of 2000 mononucleated cells were counted per dose per replicate using the principles previously established by Fenech *et al.* [41] and in accordance with the OECD guidelines [42].

### Liver functionality assays

The 3D HepG2 spheroids were assessed for liver functionality by measuring albumin (BCG Albumin Assay Kit, Merck, UK) and urea (Urea Assay Kit, Merck, UK) production over 14 days. Both assays were performed as per manufactures instructions in triplicate (*n* = 3). On the day of harvest, spheroids were pooled and centrifuged at 230 g for 5 min. Cell culture supernatant was collected for use with the albumin and urea assays and stored at −80°C until required.

### DNA extraction and duplex sequencing library preparation

Following repeated exposure to AA (5 and 20 μM), genomic DNA from 3D HepG2 spheroids was extracted using Qiagen DNeasy Blood & Tissue kit following the manufacturer’s instructions (Fig. 1), except for during the initial proteinase K digestion, where samples were also incubated with RNase A. One ULA plate (96 spheroids) per condition was harvested and pooled together to ensure enough DNA was collected for library preparation, this was repeated twice to achieve two biological replicates. DNA was eluted in 1× TE buffer low EDTA (10 mM Tris–HCl, pH 8.0, 0.1 mM EDTA) (Fisher Scientific, UK). Quality and quantity of DNA were ensured using NanoPhotometer NP80 Touch (IMPLEN GMBH) and the Qubit 4 fluorometer with dsDNA High Sensitivity Assay kit (Invitrogen). Once extracted samples were stored at −80°C until required.

**Figure 1:**
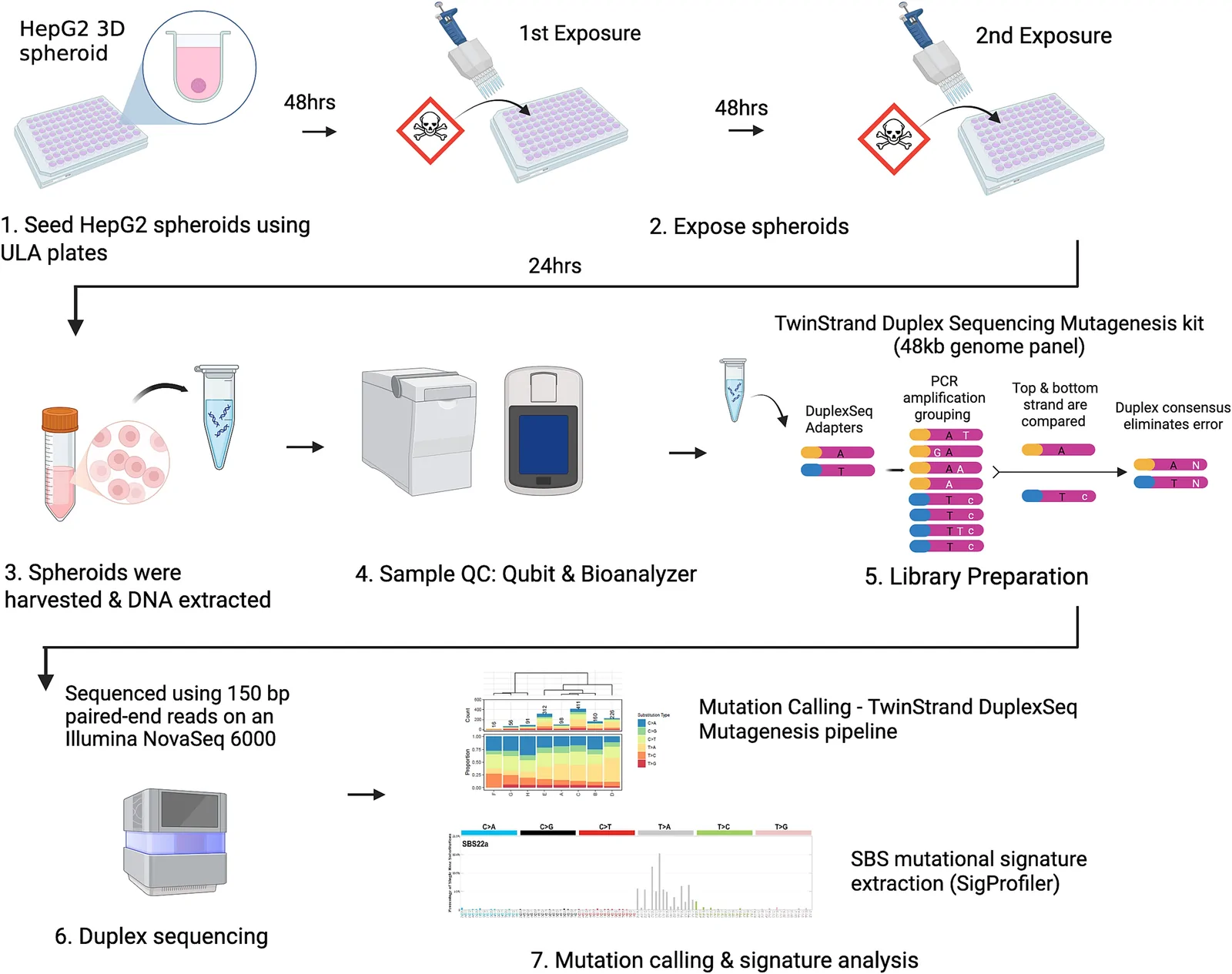
Experimental workflow for exposing HepG2 3D *in vitro* liver spheroid model to genotoxic chemicals for error-corrected DS and mutational signature analysis. Step 5 has been adapted from TwinStrand Technology [63]. Created in BioRender. Ivtg, S. (2025). https://BioRender.com/gek810f

ecNGS was performed using the TwinStrand® DuplexSeq™ Human Mutagenesis kit (TwinStrand, USA). Library preparation was performed as per the manufacturer’s instructions using a TwinStrand v1.0 Human Mutagenesis panel (a total target size of 48 kb) with DNA input sufficient to generate between 500 million and 1 billion informative duplex base pairs per sample. In short, target enrichment using the reagents and standard protocol was completed for each sample using 500 ng of DNA. DNA was enzymatically fragmented to a median size of ~300 bp, end-repaired, A-tailed, and ligated to DS adapters. The libraries were sequenced using 150 bp paired-end reads on an Illumina NovaSeq 6000. The libraries were prepared at Swansea University and sequenced at CeGaT (Tübingen, Germany).

### Mutation calling

TwinStrand Biosciences processed all raw sequencing data using the DS human mutagenesis pipeline as previously described [43]. MF was calculated by dividing the number of unique mutations (i.e. ignoring the effect of clonal expansions) by the total number of duplex bases sequenced. The TwinStrand Biosciences pipeline provides sequencing quality metrics, MF, mutation spectra, trinucleotide frequency, and MF per target data. Any mutations overlapping across samples were deemed to represent common/germline mutations and have been removed prior to signature extraction analysis. To ensure clear and robust mutation patterns for mutational signature analysis, we used stringent minimum mutation counting method.

### Mutational signature extraction and analysis

Mutation spectra of samples generated from the TwinStrand SBS calls were further filtered to retain only non-overlapping mutations, using SigprofilerMatrixGenerator (v1.2) with its R wrapper, SigProfilerMatrixGeneratorR (v1.1) 13. SBS mutational signature extraction was conducted on eight samples (four untreated time-matched controls, four AA-treated spheroids) using SigProfilerExtractorR (default parameters; v1.1.4). The optimal number of mutational signatures was assessed based on both the mean sample cosine and average stability and set to one *de novo* signature (stability of 1.0), which was subsequently decomposed into four known COSMIC signatures.

### Statistical analysis

All experiments were performed in triplicate (*n* = 3) unless otherwise stated, with data presented as mean ± standard error of the mean (SEM). Statistical analysis was performed using Prism 10, GraphPad Software, Inc. Shapiro–Wilk test was used to calculate normality for each data set. For normally distributed data, one-way analysis of variance (ANOVA) with Dunnett’s *post hoc* multiple comparison procedure was used.

## Results

### Characterization of an advanced 3D HepG2 liver model

The HepG2 cell line is commonly used for growing 3D liver cultures and for toxicological studies. Figure 2 presents an advanced HepG2 3D liver model that is both cost-effective and easy to manipulate with basic training. Spheroids were formed using ultra-low attachment plates over a 48-h period. As seen in Fig. 2A after day 3, the spheroids are circular in shape with clear and defined borders. These spheroids also show high viability (+50%) over a 14-day period demonstrating that this model can be used for both acute and chronic exposures (Fig. 2B). Additionally, we show maintained liver functionality over the 14 days. Albumin remains stable with no significant changes (Fig. 2C). Urea gradually increases from day 1 (0.5 ng/μl), peaking at day 5 (0.7 ng/μl) after which there is gradual decline until day 14 [which is significant when compared to the peak at day 5 (*P* = .006), Fig. 2D]. The metabolic competence of these spheroids was also assessed. *CYP1A1* expression was measured by real-time polymerase chain reaction and compared to HepG2 2D monocultures as demonstrated in Fig. S1. A significant increase in *CYP1A1* expression in untreated and aflatoxin B1-treated 3D spheroids compared to 2D conditions was observed, similar to previous reports [33, 44].

**Figure 2:**
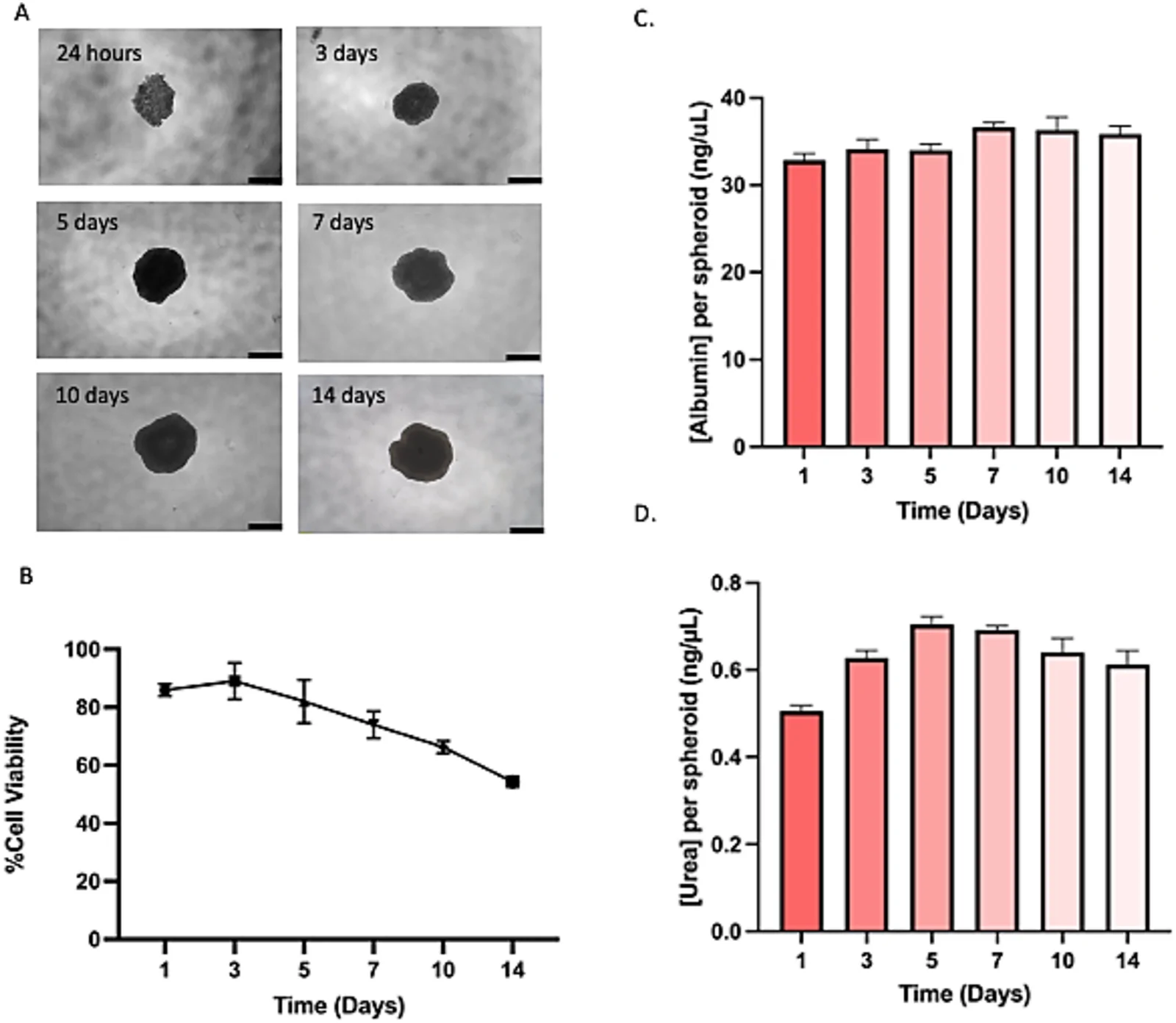
Characterization of an advanced *in vitro* HepG2 3D liver model. (A) Phase contrast images of the spheroids (4000 cells/spheroid) over 14 days, scale bar 500 μm. (B) Cell viability of the spheroids was measured by trypan blue over the 14 days. Data are presented as % viable cells ± SEM, *n* = 3. (C and D) HepG2 3D spheroid liver-like albumin and urea functionality were assessed over 14 days. Data are presented as concentration of albumin or urea per spheroid ± SEM, *n* = 3.

### Aristolochic acid induces a genotoxic response in the HepG2 3D liver model following repeated exposure

AA has been classified by the International Agency for Research on Cancer (IARC) as a Group 1 human carcinogen due to underlying carcinogenic, mutagenic, and genotoxic mechanisms [45]. While AA was originally shown to cause nephrotoxicity and urinary tract tumours, more recent studies report strong association with liver cancer [45–47], and links to kidney cancer [48–50] and cancers of multiple other organs [45, 51]. To investigate further the relevance of AA as a liver mutagen, 3D HepG2 spheroids were twice exposed as per Fig. 1 to increasing concentrations of AA over a 4-day period and analysed with the mononucleate micronucleus assay which measures fixed chromosomal damage. As spheroids grow, diffusion of oxygen and nutrients through to the centre of the spheroid is reduced and therefore it becomes necrotic. Figure 2B–D demonstrates that 3-day spheroids show higher cell viability and liver functionality than 24-h spheroids. To ensure spheroids were exposed during the early stages of spheroid development when the necrotic core was at its smallest, the first exposure to AA was at 48 h. As evident in Fig. 3A, after repeated exposure over a period of 4 days, there is a significant increase in micronuclei at 10 and 15 μM. A higher dose of 20 μM was also performed resulting in a significant genotoxic response compared to the vehicle control (*P* < .002); however, the cell viability was below the threshold (55% ± 5%) as set out by the OECD TG 487 (OECD 2023) (data not shown). As demonstrated in Fig. 3B, there is a significant drop in cell viability at 10 μM when compared to the control; however, this is less pronounced at 15 μM and is above the TG 487 cytotoxicity threshold, indicating that AA induces fixed chromosomal damage in 3D HepG2 spheroids at concentrations over 5 μM after repeated exposure.

**Figure 3:**
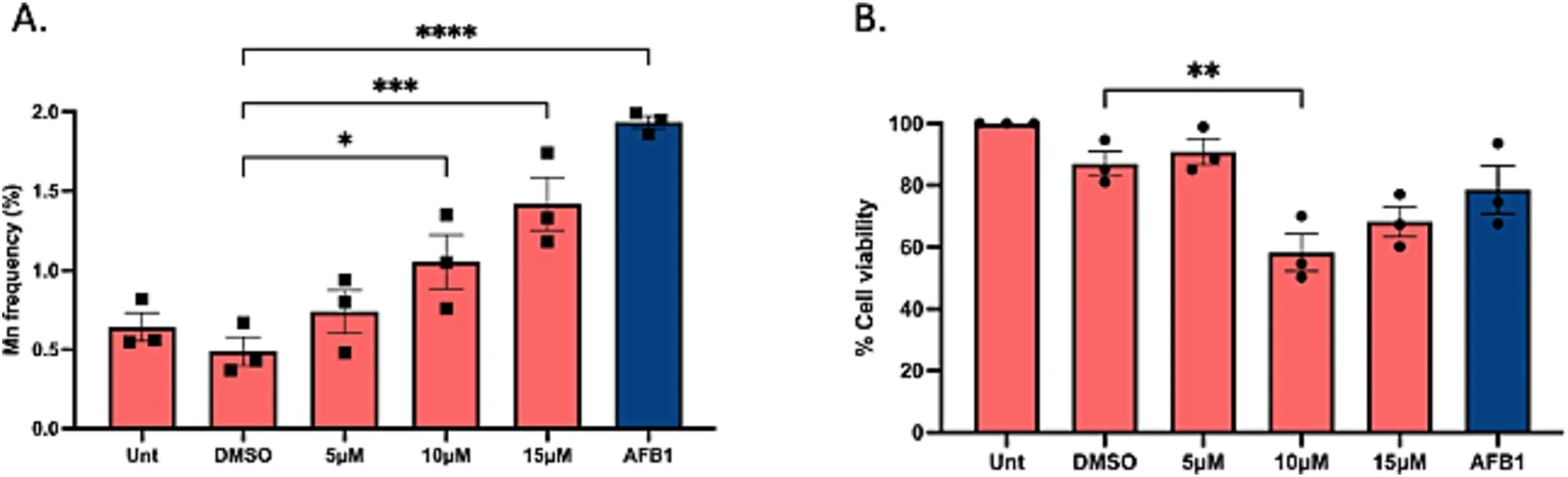
Genotoxic assessment of aristolochic acid (AA) in 3D HepG2 spheroids. Spheroids were exposed to increasing concentrations of AA (0–15 μM) for 24 h and analysed for (A) micronuclei in mononucleated cells (Mn %) and (B) relative viable cell count (RVCC). Statistical significance was compared to the untreated vehicle control and was analysed by one-way ANOVA with Dunnett’s *post hoc* analysis (^*^P < .05). Data shown are expressed as mean ± SEM, *n* = 3.

### HepG2 liver spheroids are amenable to DS-based mutational signature analysis

A workflow was developed for exposing the HepG2 3D spheroids to AA, in an effort to detect SBSs using an ecNGS/DS approach based on the TwinStrand Duplex Sequencing Mutagenesis kit (as seen in Fig. 1). As seen in Fig. 4A, there is a substantial difference in the mutation spectra and the number of substitutions detected between the untreated and AA-treated samples. We also observed an increase in the number of SBSs with increasing AA exposure concentrations. Mutational signature analysis was performed on the DS data, and the identified experimental signature was compared to the established set of COSMIC signatures. The main *de novo* extracted mutational signature identified in the sample set is shown in Fig. 4B and is predominantly characterized by T:A > A:T mutations with potential transcription strand bias for the transcribed strand suggesting adenine damage characteristic of AA effects [45]. As shown in Fig. 4C, this signature can be further decomposed into COSMIC signatures SBS40, SBS22, SBS1, and SBS5. The contribution of mutations supporting SBS40 and SBS22 is considerable (>54% and > 41% of attributed mutations, respectively), while SBS1 and SBS5 contribute very little to the experimentally derived signature (3.3% and 1.3% of all attributed mutations, respectively). SBS1 and SBS5 mutations were only attributed to the untreated samples, while SBS40 is present in both treated and untreated HepG2 spheroids (Fig. 4D), suggesting that neither of these signatures is associated with AA exposure. On the other hand, SBS22 mutations are only detected in AA-exposed samples, linking it directly to the treatment (Fig. 4D).

**Figure 4:**
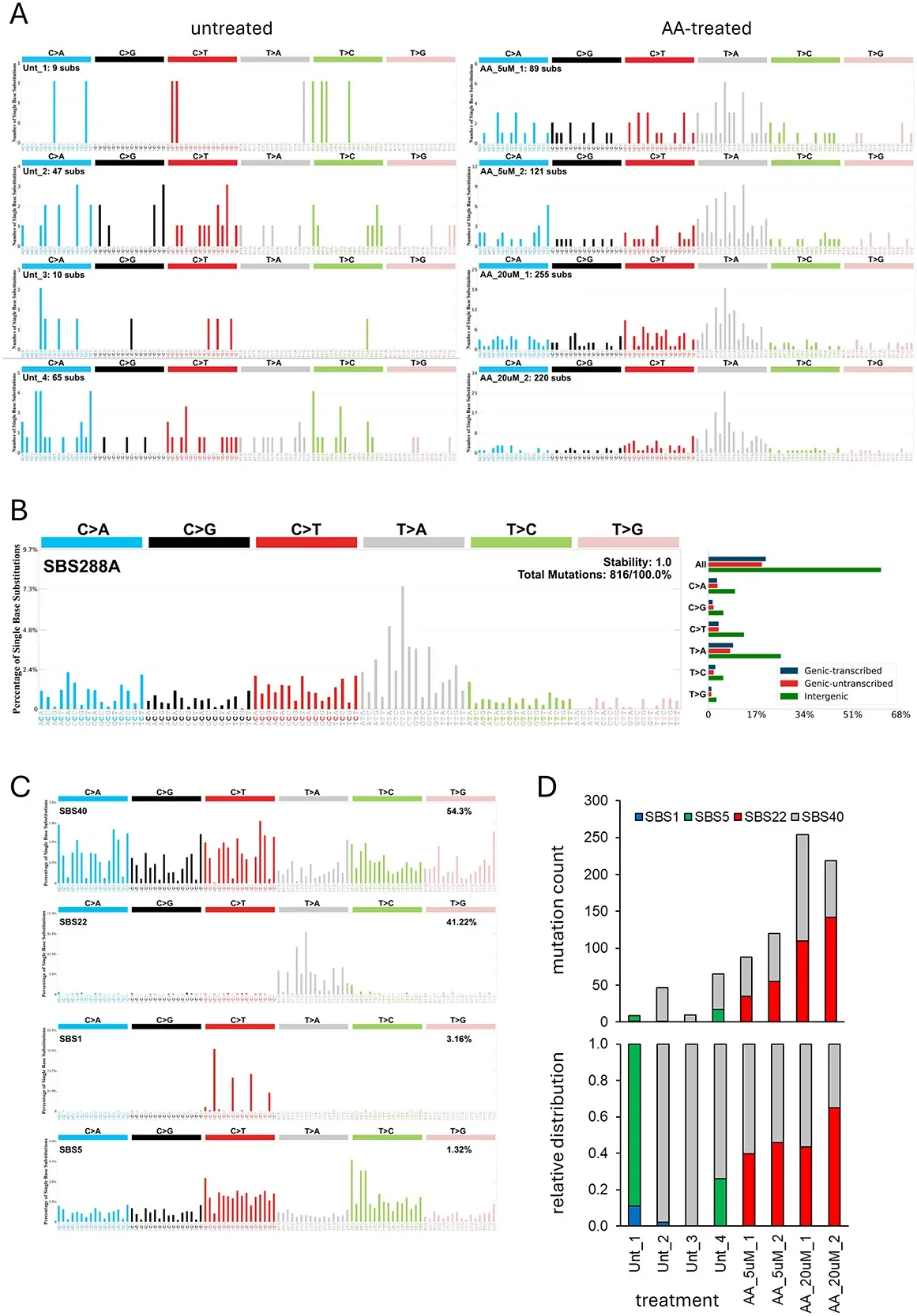
Mutation spectra and mutational signature analysis in AA-induced HepG2 spheroids. (A) Filtered TwinStrand ecNGS-generated mutation spectra of individual untreated (left panels) or AA-treated (right panels) HepG2 spheroids. AA concentrations and numbers of resulting mutations (subs) are indicated. (B) The principal *de novo* 288-channel mutational signature derived from the spectra shown in (A). The bar graph to the right indicates the proportions of signature-attributed mutations in genic transcribed/untranscribed regions, and in intergenic regions. (C) The decomposition of the 288-channel signature shown in (B) into COSMIC signatures, including SBS22, the signature of aristolochic acid exposure in human cancers. (D) The mutation counts (upper panel) and proportionate distribution (lower panel) attributed to mutational signatures shown in (C).

## Discussion

Genotoxicity testing is a fundamental part of chemical hazard assessment. However, the current testing strategy is expensive, laborious, and can be slow, in addition to the fact that many of the tests require the use of animals. The current testing strategy for assessing point mutations as set out by the OECD Test Guidelines (TG) includes the following: TG 476; *In vitro HPRT* (hypoxanthine-guanine phosphoribosyltransferase) gene mutation test, TG 490; mouse lymphoma L5178Y *TK*^+/−^ and TK6 *TK*^+/−^ assays, TG 488; *In vivo* transgenic rodent gene mutation test, TG 471; bacterial reverse mutation (or Ames test). These tests have served their purpose well and have been effective in the hazard assessment of chemicals. However, with a push from the NC3Rs to reduce, refine, and replace animals in scientific experimentation, and the requirement for the implementation and utilization of more robust *in vitro* models, the current mutagenicity testing strategy could be considered outdated and in need of modernization. Our data demonstrate a 3D HepG2 liver spheroid model that is easy to use, can be grown over 14-day period, and can be used to assess not only fixed DNA damage (micronucleus assay) but also point mutations using advanced DS technology. ecNGS/DS offers a high sensitivity and in-depth characterization approach of chemical-induced mutagenesis [52]. An advantage of using DS is that it can be performed in any cell type, requiring a very small quantity of cellular DNA and can yield results quicker than the traditional battery of tests. In addition, and compared to the current standard of tests, DS has the capacity to assess mutagenicity in any region of the genome or on any gene of interest [7]. However, convincing regulators to move to alternative methods requires building a body of evidence to support its use.

To date, there is limited published data utilizing DS in *in vitro* models, particularly in liver cells. Traditionally, when preparing *in vitro*-based samples for whole genome sequencing (WGS), cells would have to undergo clonal expansion, which increases the possibility of monoclonality to generate a homogenous population of cells derived from a single parent cell. This enables the detection of mutations caused by genotoxic compounds using standard NGS approaches [53]. Examples of these techniques include single-cell subcloning expansion and barrier-bypass clonal expansion [26, 54–56]. Traditional methods of single-cell isolation and clonal expansion are cost effective and convenient; however, they are laborious and very time consuming, as they are based on the probability of Poisson distribution to distribute one cell per well [57]. Novel NGS approaches such as DS have the accuracy to detect low-frequency mutations, negating the need for clonal expansion [9], therefore, drastically reducing the workflow required. Recently, DS was used to detect and quantify mutations in human lymphoblastoid TK6 cells in an inter-laboratory trial. While being a single-cell suspension system, TK6 cells are widely used in regulatory genotoxicology. The authors highlighted the consistency of the results both across time and between the laboratories. Interestingly, the authors identified that for cells sampled between 48 and 96 h following initial exposure, both laboratories demonstrate similar over all mutation frequencies, induced mutation frequencies and proportions of base substitution types [58]. This is a significantly reduced timeframe when compared to the HPRT assay, which can take 2–3 weeks [58]. Wang *et al.* demonstrated the efficacy of using DS to quantify mutagenesis using an organotypic human air–liquid interface airway tissue model following exposure to ethyl methanesulfonate [59]. The advanced airway model used in this study is composed of three cell types (ciliated cells, goblet cells, and basal cells) typically found in the low large airway of humans, retaining the functional characteristic of the human epithelial tissue. The authors noted difficulties in utilizing traditional genotoxicological methods such as the micronucleus assay, *HPRT*, and thymidine kinase *(TK)* due to the fact that the cell populations are largely differentiated and not dividing, the requirement for disaggregation into single cells, and potential requirement of producing single-cell clones [59]. As a result, the authors employed DS to demonstrate the efficacy in advanced models. DS was also successfully employed to assess DNA damage and mutagenic effects of ultraviolet radiation using primary mouse embryonic fibroblasts (MEFs), human foreskin fibroblasts (HFFs), and adult human epidermal keratinocytes (HEKa) [56]. The authors performed standard WGS following clonal expansion (MEF and HFF) and DS without clonal expansion (HEKa). They noted that cells that underwent clonal expansion can demonstrate high background mutations due to genomic instability [56]. Most recently, Seo *et al.* have demonstrated that HepaRG liver cells in both 2D and 3D cultures can be used for mutation assessment measuring fixed DNA damage and mutagenesis using DS, micronucleus, and the comet assay [60]. Interestingly, the authors noted that the most commonly used DS technology only sequences 48 kb of the human genome and requires large cell numbers to achieve sufficient mutation numbers. Therefore, mutational frequencies and mutation spectra might be influenced by cytotoxicity and the stringency of mutation calling. They observed the same mutation spectra from non-toxic concentrations of NDMA in both 2D and 3D HepaRG cells, hypothesizing that the higher toxic concentrations reduced the cell population to the point where mutations could not be detected [60].

Similarly to these studies, our data demonstrate that DS and mutational signature analyses can detect and quantify differences in the number of substitutions between AA-treated and untreated samples. Typically, conversion of DNA lesions into mutations typically requires multiple rounds of cell division, therefore, when performing ecNGS with *in vitro* samples, it is important to consider population doubling time and ensure continued cell proliferation during experimental design. For this study, to ensure mutations were fixed, cells were exposed to AA twice, with a 48-h period between the first and second exposure and a further 24 h after the second exposure prior to harvesting. This approach was successful as demonstrated by the detection of SBS22 signature. Our data further highlight an increase in the number of SBSs with increasing AA exposure concentrations, and we show that the *de novo* mutational signature identified is characterized by T:A > A:T mutations with transcription strand bias suggesting adenine damage. Recently, Sahib *et al.* demonstrated the efficacy of using DS to evaluate the mutagenicity of AA, *in vivo* using Fisher 344 rats [13]. The MF for the AA-treated group was increased 44-fold over the vehicle control with the primary mutation type being A:T > T:A transversions [13]. A recent study by Sun *et al.* investigated metabolic reprogramming in 12 liver cancer cell lines using whole exome sequencing and mutational signature analysis [61]. Interestingly, as seen with the data presented here, they also note the presence of SBS40 (in addition to SBS1 and SBS5) in the HepG2 cell line, moreover, SBS40 was present in 11 out of the 12 liver cell lines investigated [61]. In contrast to SBS40, SBS1, and SBS5, signature SBS22, which contributes prominently to the experimental signature (supported by > 40% of all attributed mutations), is only present in the exposed spheroids with the relative abundance reflecting the AA treatment doses. Signature SBS22 is commonly found in many cancer types in patients exposed to AA [14, 45, 51]. Interestingly, while the micronucleus assay was unable to detect any significant fixed DNA damage at 5 μM, DS was able to identify exposure-related T:A > A:T mutations at the low dose of 5 μM. This emphasizes the sensitivity of DS over current conventional methods for detecting DNA damage at low concentrations. These data highlight the suitability of *in vitro* HepG2 spheroids for evaluating mutagenicity at the level of mutational signatures using alternative approaches such as DS.

DS is an expensive technique that requires specialist training and access to a sequencer to generate high-quality data. However, over the past decade, the costs of performing NGS have been reducing significantly, making DS a more generally accessible, interesting option for mutagenesis studies [43]. In addition to analysing and comprehending big data, rigorous bioinformatic approaches are required to be performed by highly trained personnel. Moreover, prior to being implemented into the current mutagenicity testing strategy, there is also a need to standardize the library preparation and bioinformatic pipeline to ensure consistency in the data and analysis across the field. DS can detect SBSs and small indels, however, unlike the currently used *HPRT* and cell gene mutation test using the *TK* gene, it cannot detect large deletions and chromosomal rearrangements [58]; therefore, it is unlikely to replace current methods rather than be added to the testing approach.

The current battery of tests for evaluating mutagenicity *in vitro* have served us well; however, as mentioned previously, they do suffer from limitations. One of the main constraints being that the mammalian cell mutation assays are limited to 2D monocultures and are usually restricted to the evaluation of a single gene. It is now widely accepted that 2D systems have several limitations and do not adequately simulate an *in vivo* environment [30, 31]. It is important that we continue to improve the current testing strategy to include the use of new technologies and advanced *in vitro* models such as 3D spheroids, and co-culture systems that enhance the physiological relevance of the data, as they better represent the human system. In addition, advanced *in vitro* systems provide robust data with mechanistic insights, supporting the 3R’s initiative, to reduce the use animal models in experimentation. In conclusion, linking the exposure of specific toxins to mutagenesis and carcinogenesis remains in its infancy; however, the aforementioned studies and the data we have provided demonstrate the power of ecNGS/DS and mutational signature analyses for measuring AA-induced DNA damage in an advanced 3D HepG2 liver model.

## Supplementary Material

Supplement_Figure_1_geaf015

## Data Availability

The authors will freely release all data underlying the published paper upon direct request to the corresponding author.
