# Supplementary material for: Harnessing the power of an advanced in vitro 3D liver model and error-corrected duplex sequencing for the detection of mutational signatures
Source: Mutagenesis. 2025 Aug 6;41(6):320–9. doi: 10.1093/mutage/geaf015 (PMC13711086; doi:10.1093/mutage/geaf015)
Supplement: Supplementary file 1 [file supplement_figure_1_geaf015.docx]

Supplement Figure 1

SF1. Assessment of metabolic capacity of 3D HepG2 spheroids 5 days post seeding.

Methods

Assessment of metabolic capacity of 3D HepG2 spheroids was carried out by quantitative RT-PCR. 2D monolayers and 3D HepG2 spheroids were treated with 0.4uM Aflatoxin b1 for 24hrs. Cells were harvested as described in the methods of the main text. Total cellular RNA was extracted using an RNA preparation kit (RNeasy Mini Kit, Qiagen) and the subsequent, cDNA was synthesised using iScript cDNA Synthesis kit, Bio-Rad. qPCR master mix was prepared using Bio-Rad SYBR green and primers as detailed below and run on Bio-Rad CFX connect. *HPRT1* and *B2M* were used as reference genes. Analysis was performed using Bio-Rad CFX Maestro software and PRISM GraphPad as described previously.

| Gene | Sequence |
| --- | --- |
| *B2M* Forward | GTTTACTCACGTCATCCAGC |
| *B2M* Reverse | AGACAAGTCTGAATGCTCCA |
| *HPRT1* Forward | TGATGTAATCCAGCAGGTCA |
| *HPRT1* Reverse | ACTGAACGTCTTGCTCGAGA |
| *CYP1A1* forward | GCACAGAGGTAGTCTCACTGCTTG |
| *CYP1A1* reverse | AAGGGCAGAGGAATGTGATGTT |
